# Primary central nervous system post-transplant lymphoproliferative disorders: the spectrum of imaging appearances and differential

**DOI:** 10.1186/s13244-019-0726-6

**Published:** 2019-04-11

**Authors:** Matthew L. White, Drew W. Moore, Yan Zhang, Keiper D. Mark, Timothy C. Greiner, Philip J. Bierman

**Affiliations:** 1 Radiology, University of Nebraska Medical Center, 981045 Nebraska Medical Center, Omaha, NE 68198-1045 USA; 2 Pathology, University of Nebraska Medical Center, 983135 Nebraska Medical Center, Omaha, NE 68198-3135 USA; 3 Oncology, University of Nebraska Medical Center, 986840 Nebraska Medical Center, Omaha, NE 68198-6840 USA

**Keywords:** Central nervous system, Post-transplant lymphoproliferative disorders, MRI, CT, Clinical features

## Abstract

**Objective:**

Central nervous system post-transplant lymphoproliferative disorder (CNS-PTLD) is a rare disease that presents with non-specific signs and symptoms. The purpose of this article is to present the imaging appearances of CNS-PTLD by magnetic resonance imaging. We highlight the differential diagnostic considerations including primary central nervous system lymphoma, glioblastoma, cerebral abscess, and metastatic disease. This is an important topic to review since in daily practice the diagnosis of CNS-PTLD is often not initially considered when present due to its rarity and the lack of radiologists’ familiarity with the disease.

**Conclusion:**

Knowing the unique imaging features of CNS-PTLD narrows the differential diagnosis, facilitates the diagnostic work-up, and optimizes making the diagnosis. Advanced MRI data for CNS PTLD is limited but is promising for helping with narrowing the differential diagnosis.

## Key points

- There is a tendency for CNS-PTLD lesions to be ring enhancing, have ill-defined enhancing margins, multifocal, supratentorial, and lobar in location.
- The solid areas of CNS-PTLD lesions often have restricted diffusion likely related to hypercellularity.
- Perfusion analysis would demonstrate a lower maximum relative cerebral blood volume in CNS-PTLD.
- CNS-PTLD shares conventional imaging characteristics with multiple other disease processes including PCNS lymphoma, GBM, metastatic disease, abscess, and other infections.
- It is important to know the clinical history and to consider the diagnosis of CNS-PTLD to facilitate the diagnosis.

Central nervous system post-transplant lymphoproliferative disorder (CNS-PTLD) is a rare disorder due to immunosuppression secondary to solid organ, stem cell, or bone marrow transplantation [1, 2]. Cases begin to appear as early as 6 weeks in the first year after transplantation but most cases take years to present after transplantation and the attendant immunosuppression. Viral factors have been strongly associated with CNS-PTLD and the strongest association is with Epstein-Barr virus (EBV). The neurological symptoms that patients present with are quite variable. The symptoms are related to general neurological dysfunction as well as specific signs and symptoms related to the location of the CNS lesions. Early consideration for CNS-PTLD is paramount to institute an appropriate work-up and to determine the needed therapy. Since an early step in the work-up is often neuroimaging, a radiologist aware of the imaging characteristics of CNS-PTLD and the differential diagnosis can positively impact patient care.

CNS-PTLD cases start presenting within the first year after the immunosuppression with a median time to occurrence around 4–5 years [3–7]. However, there are late occurring cases greater than 10 years after the initial transplantation [3–7]. PTLD occurs more frequently in the first year after transplant in patients that are EBV-positive after transplantation, young, EBV-negative prior to transplant, have multi-organ transplants, and have allograft involvement [3–9]. Earlier occurring cases are very likely to be diffuse large B cell lymphoma. With long-term follow-up beyond 10 years, more cases are EBV-negative, T cell lymphomas (especially in renal transplant patients), and there is an association with hepatitis C infection [1, 3–7]. Overall, the vast majority of all cases are diffuse large B cell lymphomas [3]. Old age is associated with later onset PTLD and EBV-negative cases that are more frequently monomorphic. PTLD tends to occur more in children than adults with no gender preference. The incidence of PTLD after solid organ transplant varies by organ. Greater incidences are generally associated with small bowel, lung, and heart/lung, and lower incidences with heart, liver, pancreas, and kidney transplantation [1, 3, 4, 6–8, 10]. Kidney transplant recipients have the highest absolute number of PTLDs which is secondary to the higher number of kidney transplants performed. The difference in incidence among transplant types is likely related to the different immunosuppressive regimes and the amount of EBV-positive lymphocytes in the graft. Also, differences in underlying transplanted organs might confer different levels of chronic antigen stimulation relevant to lymphomagenesis. CNS-PTLD accounts for between 5 and 30% of all PTLD cases. CNS-PTLD patients tend to have poorer survival than patients with PTLD outside of the CNS but given the paucity of CNS-PTLD cases, this analysis is limited. EBV-associated lymphoproliferative disorders can occur in other forms of immunosuppression such as with acquired immune deficiency syndrome (AIDS), and these cases have similar imaging characteristics (Table 1).

**Table 1:** MR imaging features CNS PTLD vs AIDS CNS Lymphoma and PCNS lymphoma

	CNS PTLD	AIDS CNS Lymphoma	PCNS lymphoma
Clinical	Status post-transplant of solid organ, stem cell, or bone marrow	HIV positive patient	Elderly patient, Wiskott-Aldrich syndrome, ataxia-telangiectasia, severe-combined or common-variable immunodeficiency, rheumatoid arthritis, and systemic lupus erythematosus
Lesion number	Multifocal more common than unifocal	Multifocal	Unifocal more common than multifocal
Lesion location	Lobar predominantly, numerous basal ganglia and thalamic lesions, less commonly abuts CSF surface	Basal ganglia and corpus callosum	Periventricular, abutting CSF surface
Enhancement pattern	Ring	Ring	Solid
Enhancement	Heterogeneous	Heterogeneous	Homogeneous
Margin of enhancement	Irregular/Ill-defined	Irregular	Well-defined
ADC	Elevated compared to lymphoma, will still have focal areas of restricted diffusion	Slightly elevated compared to normal white matter but lower than in toxoplasmosis	Lower and more homogeneous
Perfusion	Limited data, likely overall low (Fig. 2)	Not reported	Low to mildly elevated, leakage pattern very suggestive
Spectroscopy	Increased choline, lipid and lactate Decreased NAA	Increased choline Decreased NAA, Cr	Increased choline, lipid and lactateDecreased NAA, Cr
Intratumoral susceptibility signal	Peripheral pattern of punctate hypointensities, tendency to bleed	Not reported, there is a tendency to bleed more than PCNS lymphoma	Minimal signal changes

The neurological symptoms in CNS-PTLD can be nonspecific and are quite variable. The non-specific symptoms include headache, confusional states/mental status changes, and seizure [3, 4]. Headaches are reported as a more common symptom in some studies [3–5]. Also, seizure can present with focal symptoms (e.g., motor, sensory, vision, or memory changes) indicating the lesion location. Reported focal neurological symptoms include hemiparesis, difficulty walking, ataxia, aphasia, dysarthria, facial droop, vertigo, and diplopia [3–5].

It is possible to make the diagnosis of CNS-PTLD with cerebral spinal fluid (CSF) sampling only. Positive CSF PCR for EBV is highly suggestive of the diagnosis and the CSF PCR for EBV can be positive even when PCR on the peripheral blood is negative [11]. Caution is advised in evaluating the CSF by PCR when the blood is positive for EBV. CSF cytological analysis is usually positive in fewer cases but in some small series, a majority of patients have positive CSF cytology [3–5, 7]. Flow cytometric analysis can assist in making the diagnosis of PTLD in the CSF. However, overall, the CSF analysis including CSF cytological analysis usually results in non-specific findings. Biopsy is almost always required for a definitive diagnosis [12].

## Pathology

**Fig. 1:**
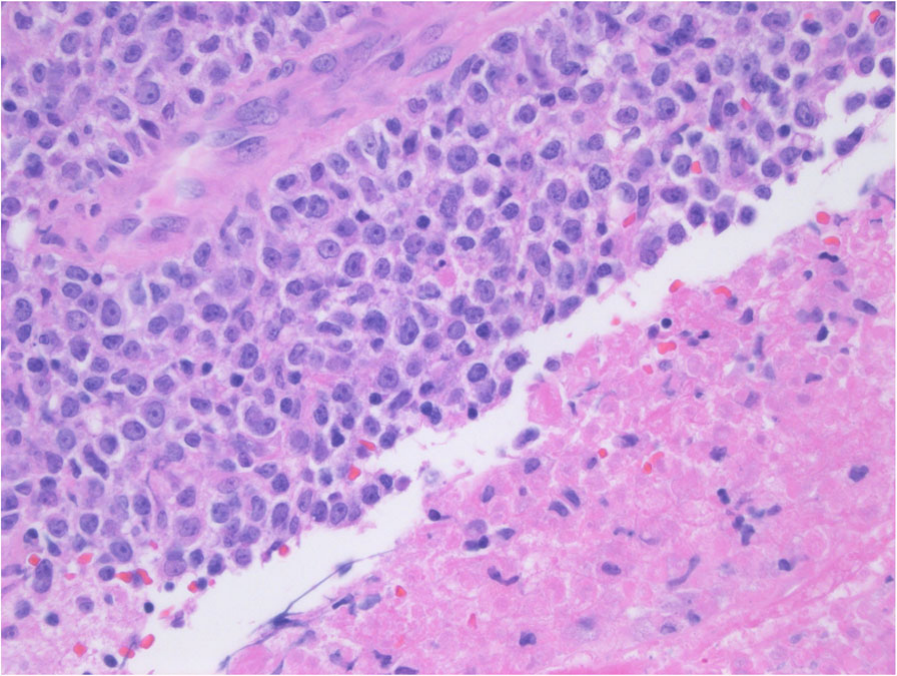
Monomorphic primary central nervous system post-transplant lymphoproliferative disorder. Photomicrograph of the specimen shows sheets of large cells which are adjacent to an area of coagulation necrosis, a common finding in biopsies of primary central nervous system post-transplant lymphoproliferative disorder. This monomorphic case could also be called diffuse large B cell lymphoma

CNS-PTLD cases are usually monomorphic by WHO criteria but some cases are polymorphic. Monomorphic CNS-PTLD usually resembles aggressive diffuse large cell lymphomas, nearly always with a B cell phenotype (Fig. 1). CNS-PTLD has a predisposition for growth in the perivascular spaces. These are more common of non-germinal center cell origin, known as the activated B cell-like origin, a subtype that has a worse prognosis. CNS-PTLD of T cell lineage is rare and is seen more frequently in sites outside of the CNS.

## Treatment and survival

The survival is quite variable with some patients dying within the first month but many patients survive for years [3–7]. The treatment almost invariably involves decreasing or withdrawal of immunosuppressive therapy in the first instance [3, 4, 7, 12]. This must be balanced with not causing rejection of the transplanted organ. Corticosteroids are used routinely. Treatment with chemotherapy or radiotherapy should be strongly considered and the therapies utilized have been rather heterogeneous [3, 4, 7, 12]. However, after multivariate analysis, Cavaliere et al. only found age to be predictive of survival [3]. Methotrexate has more commonly been used intravenously with less common utilization intrathecally [3, 4, 12]. There have also been promising results utilizing rituximab with cranial radiation [13]. There is a trend for improved progression-free survival in patients receiving rituximab and/or cytarabine [7]. More patients are being treated with antiviral therapies with mixed results. Mortality is very high in non-responders to immunosuppressive therapy modification and failures of treatment with first modality [7].

## Imaging

Identifying the imaging characteristics of CNS-PTLD can aid in the diagnosis and help differentiate CNS-PTLD from several differential diagnostic considerations. A neuroimaging exam can be used to detect the lesion(s), demonstrate the neuroanatomical areas involved, and associated complications such as brain herniation. The MRI or CT examinations should be performed with and without intravenous contrast. Contrast greatly helps to define the lesions in CNS-PTLD. MRI is superior to CT in sensitivity and for detailed analysis of CNS-PTLD lesions. An MRI protocol to best delineate and characterize the brain lesions should include T2-weighted/T2 fluid-attenuated inversion recovery (FLAIR), T1-weighted pre contrast, a gradient-echo or susceptibility-weighted imaging sequence, diffusion-weighted/diffusion tensor imaging (DWI), and the post-contrast T1-weighted images. Perfusion-weighted imaging (PWI) should also be useful to help differentiate CNS-PTLD from other potential diagnostic considerations.

**Fig. 2:**
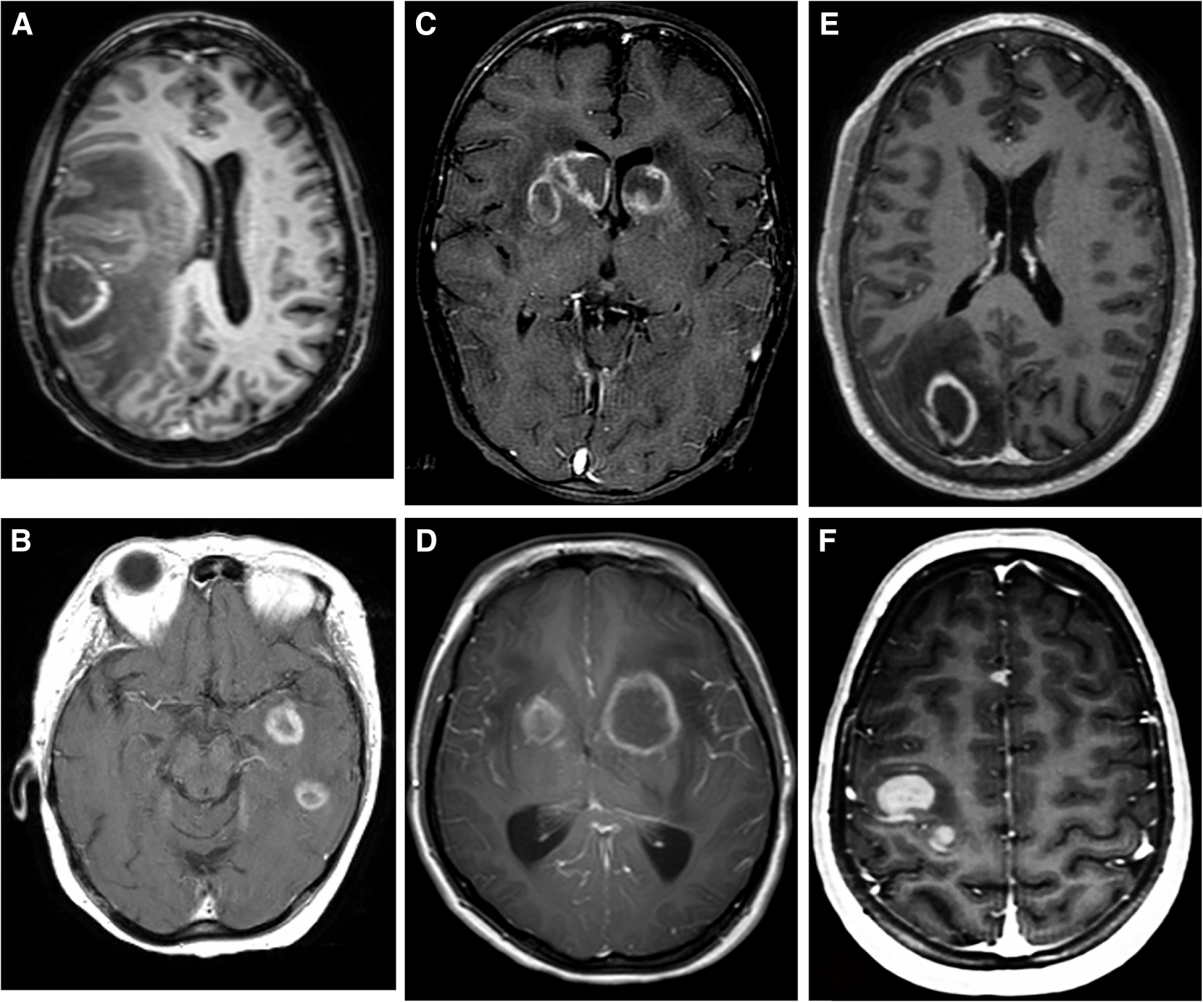
CNS-PTLD T1-weighted post-gadolinium images in six cases (**a**-**f**). In these cases, the lesions are predominantly ring enhancing. Although, case **f** has two solid enhancing lesions. There are no solid enhancing periventricular lesions as typically seen in CNS lymphoma. The location of lesions in the basal ganglia in cases **c** and **d** would be unusual for metastatic disease

**Fig. 3:**
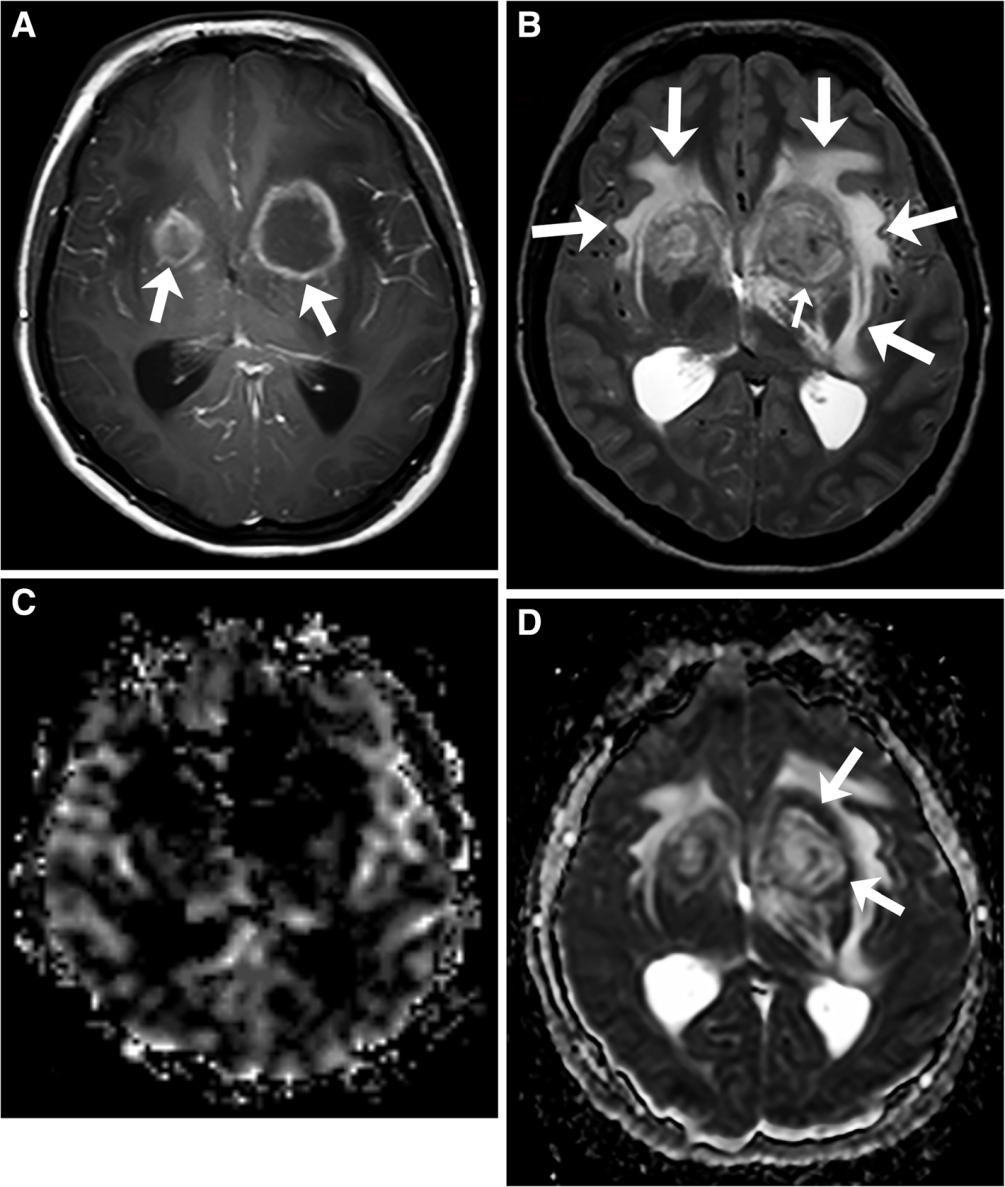
Primary central nervous system post-transplant lymphoproliferative disorder. T1-weighted image with gadolinium (**a**) shows multiple ring enhancing masses (arrows) in the basal ganglia. T2-weighted image (**b**) shows extensive edema (arrows) and mild T2 isointense rims (small arrow). CBV map (**c**) demonstrates no increased perfusion. ADC map (**d**) shows restricted diffusion of the ring enhancing area (arrows) and elevated diffusion in the central area

There is a tendency for CNS-PTLD lesions to be ring enhancing, have ill-defined enhancing margins, multifocal, supratentorial, and lobar in location (Figs. 2 and 3) [3–6, 12, 14]. The ring enhancement is secondary to central necrosis, which is a frequent microscopic finding. A partial ring of enhancement appearance has been described [5]. Diffuse enhancement in general is less common but Cavaliere et al. in a large series of 34 patients reported a higher percentage with homogeneous enhancement (41%) or heterogeneous enhancement (56%) and only 29% had ring enhancement [3, 14]. Snanoudj et al. described 87% of cases to have at least one ring-enhancing lesion and all cases to have lesions with enhancement [12]. The contrast enhancement characteristics have not always been mentioned [4, 6, 7].

Numerous authors have found lesions to be multifocal with predominantly lobar location [3, 5, 6, 12]. Predominant multifocal disease has been reported in 61% to 88% cases [3, 5, 6, 12]. An exception is Evens et al. who reported that 63% of cases had one lesion [7]. There is a greater amount of variability with the described location of CNS-PTLD lesions. Evens et al. described a minority of cases to have deep involvement of the basal ganglia, brainstem, cerebellum, and/or periventricular involvement [7]. However, the basal ganglia can be frequently involved and even the second most common location (39%) [3, 6]. Although, others have found the periventricular location to be the second most common location and at least one periventricular lesion being seen in 72% of cases [5, 12]. Usually lesions are less commonly reportedly in the corpus callosum, thalami, and periventricular locations [4–6]. Rarely, lesions are present in isolation in non-lobar locations including infratentorially or in the spinal cord. Rare presentations are generally found to include cerebral lymphomatosis and associated or isolated meningeal contrast enhancing lesions [4]. However, there has been concurrent meningeal enhancement reported in over 50% of patients and meningeal/ependymal enhancement in 40% of patients [3, 5].

The CT density of CNS-PTLD lesions has a wide reported variation. Lesions on CT have been reported to be hyper, iso, or hypoattenuating [12, 15, 16]. The presence of hypodensity in the lesions differs from what has been described for PCNS lymphoma [17, 18]. However, mild hyperdensity might be present due to hypercellularity or to the presence of hemorrhage [16, 19].

**Fig. 4:**
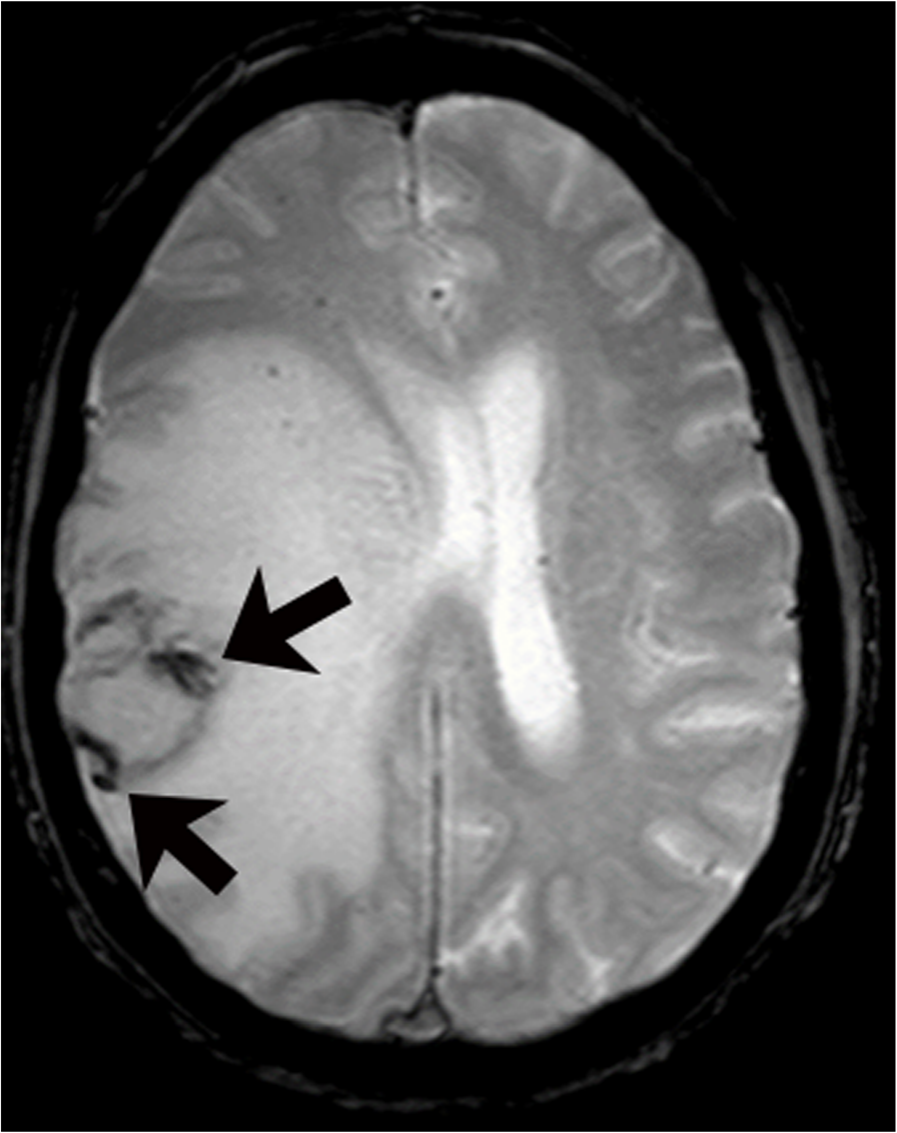
Primary central nervous system post-transplant lymphoproliferative disorder. Gradient echo image shows a mass is in the right parietal lobe with surrounding vasogenic edema. Peripheral greater than central low gradient echo signal (arrows) within the mass are suggestive of hemorrhage

The data on MRI appearance of CNS-PTLD lesions is limited. The imaging findings reflect lesions that are hypercellular tumors, which are prone to hemorrhage, have cystic and necrotic changes, and surrounding edema. T1-weighted signal intensity has been reported as predominantly hypointense to isointense with few cases having hyperintense or heterogeneous T1 signal [5, 12, 16]. The T1-weighted hyperintense signal likely represents hemorrhage. The solid areas of the masses have relative T2-weighted hypointensity related to hypercellularity but intermediate and increased T2-weighted signal intensities are also seen [16, 19–21]. The cystic and necrotic areas have signal intensities that follow fluid. Given the usually intermixture of solid areas with cystic/necrotic changes, the T2 signal intensities of the lesions are mixed. The T2-weighted images demonstrate well the surrounding edema and the edema around the CNS-PTLD lesions vary from mild to extensive (Fig. 3) [5, 19]. Typically, there is mild edema around small lesions and extensive edema around larger lesions. Susceptibility weighted imaging demonstrates a peripheral pattern of punctate hypointensities that can help discriminate CNS-PTLD from de novo PCNS lymphoma (Fig. 4) [14]. Evidence of hemorrhage or hemosiderin staining was found in five of nine by Lake et al. with one hemorrhage being significant [5].

**Fig. 5:**
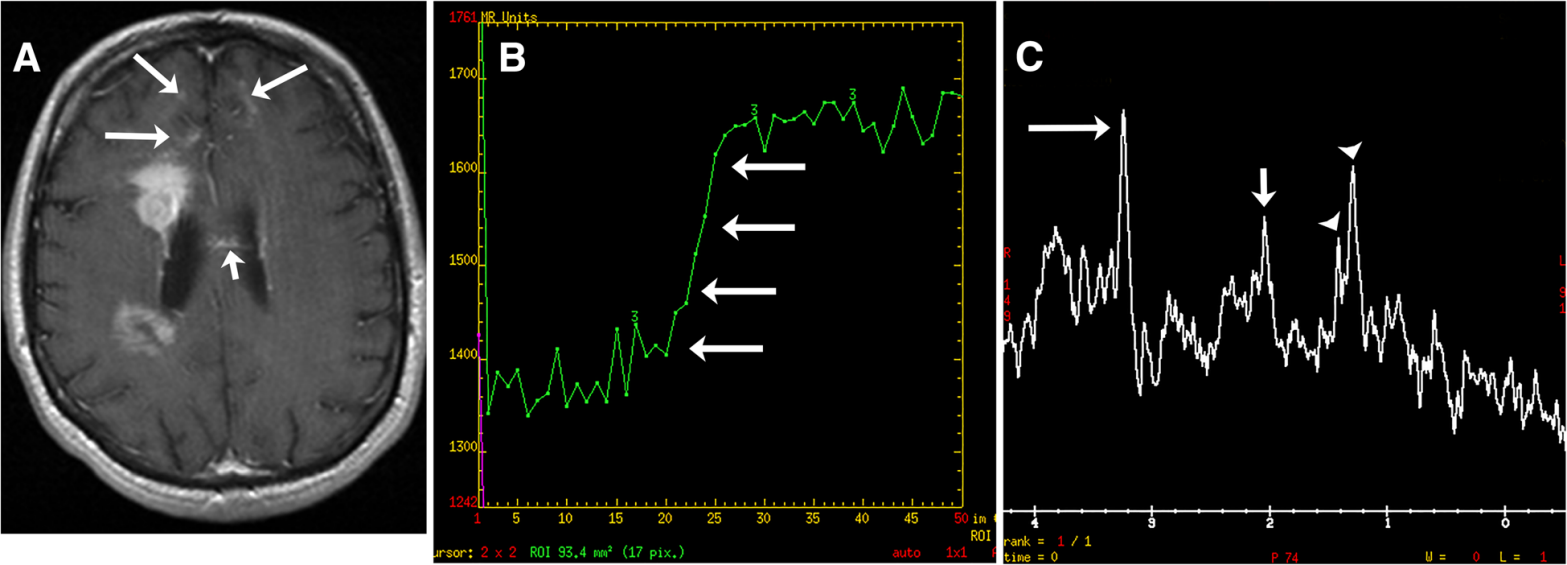
Primary central nervous system lymphoma. T1-weighted image with gadolinium (**a**) shows two lesions with intense enhancement along the right lateral ventricle. Also, note the frontal subcortical enhancement (long arrows) and linear enhancement in the corpus callosum (short arrow). Perfusion curve (**b**) shows increasing signal without the expected signal drop on echo-planar T2* perfusion due to gadolinium leaking thru a disrupted blood-brain barrier (arrows). Single voxel spectroscopy (TE = 35) (**c**) demonstrates elevated choline (long arrow) due to increased cellularity, decreased NAA (short arrow), and presence of lipid/lactate (arrowheads). No creatine peak is present

The solid areas of the lesions often have restricted diffusion likely related to hypercellularity. The diffusion is elevated in the cystic necrotic areas (Fig. 3). This results in appearance with rims of the lesion that have restricted diffusion and scattered areas of restricted diffusion internally where there are areas of hypercellularity (Fig. 3). The apparent diffusion coefficient (ADC) values in CNS-PTLD vs CNS lymphoma are on average higher and more heterogeneous [14]. There is limited literature on gadolinium-based perfusion imaging in CNS-PTLD but low relative CBV ratios have been reported (Fig. 3) [22]. The MR spectroscopic data is very limited for CNS-PTLD [23]. The solid area of enhancement demonstrated elevated choline (Cho), and marked increases in lactate and lipid [23]. In the image from the article by Su et al., the *N*-acetylaspartate (NAA) is markedly decreased but the authors describe it as preserved [23]. The creatinine (Cr) level is hard to analyze given no normal comparison but has a low appearance. These changes did result in elevated Cho/Cr and decreased NAA/Cho ratios (Fig. 5).

**Fig. 6:**
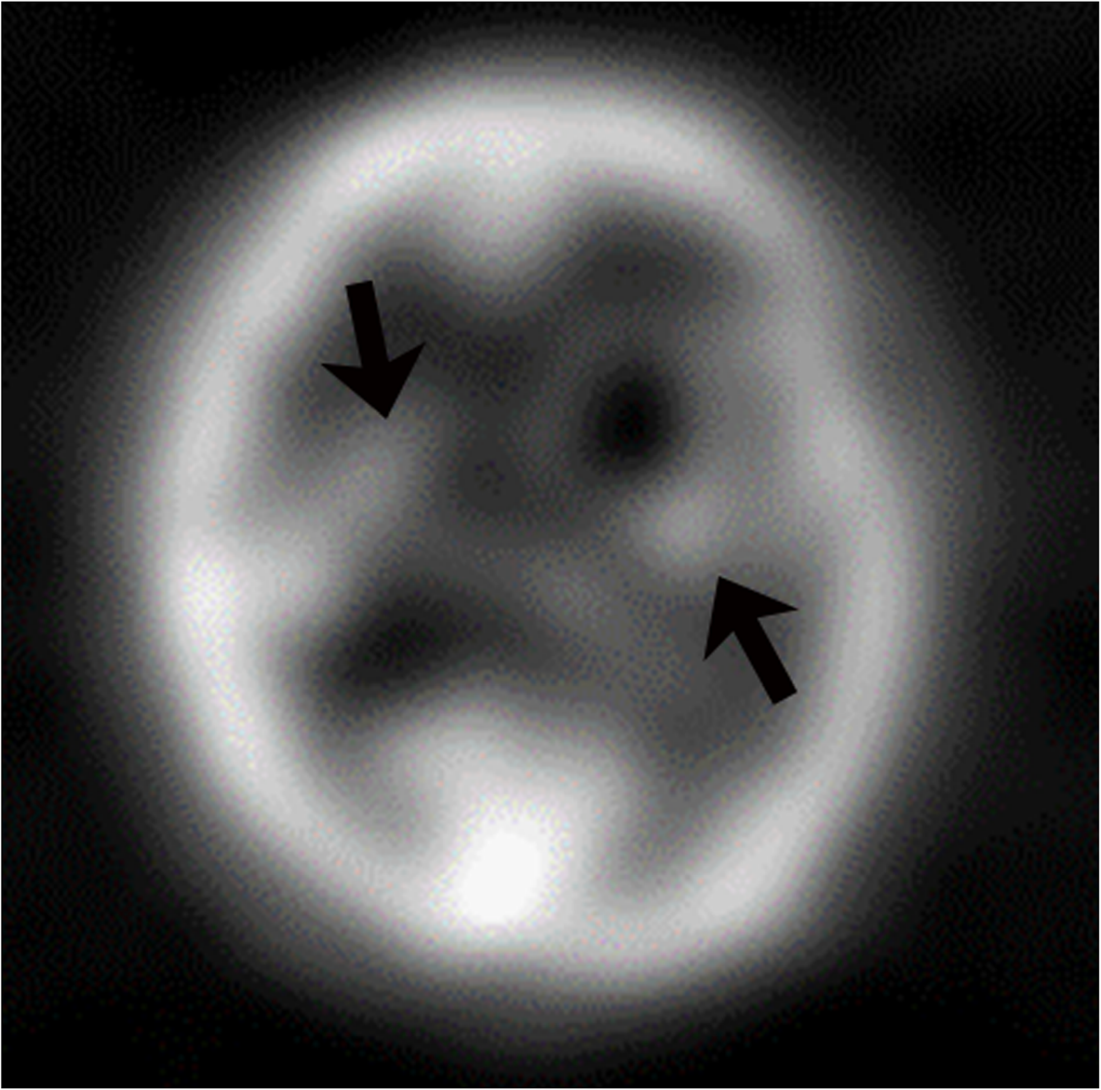
Primary central nervous system post-transplant lymphoproliferative disorder. F-18 FDG PET exam demonstrates bilateral cerebral hypermetabolic lesions in the basal ganglia (arrows). The lesion on the left is larger but the lesion in the right basal ganglia had a higher SUVmax of 8.5 versus SUVmax of 7.2 in the left basal ganglia. Notice how hypermetabolic the cortex is and how this could obscure more peripheral lesions

Positron emission tomography (PET)/CT has been used to evaluate CNS-PTLD, although it may be more usefulness for identifying PTLD lesions outside the CNS. CNS lesions on PET/CT are difficult to interpret given the inherent increased uptake of the surrounding brain parenchyma. However, PTLD does show increased SUV uptake on PET, which can help to differentiate between residual tumor and necrosis even in the CNS (Fig. 6) [19]. There can be false positive findings with infectious or inflammatory etiologies. Utilization of PET/CT is likely more beneficial in the follow-up and post treatment setting.

## Differential diagnosis

The differential diagnosis includes PCNS lymphoma, glioblastoma (GBM), metastatic disease, abscess, or other infection. Infections in transplant patients can include *Aspergillus*, *Nocardia asteroides*, *Toxoplasma gondii*, *Listeria monocytogenes*, Mucorales, Tuberculosis, and less commonly Cryptococcus [24]. Less likely differential diagnostic considerations are tumefactive demyelination, stroke, or neurosarcoidosis. Imaging characteristics can help narrow the differential diagnosis (Tables 1 and 2).

**Table 2:** How to differentiate CNS PTLD from other pathologies

GBM	
◦ High perfusion in the enhancing mass	
◦ More commonly will be a single enhancing lesion, around 2/3 of the time	
◦ More common involvement of the corpus callosum	
◦ Peritumoral region can have abnormalities indicating tumor (increased perfusion, restricted diffusion, tumor spectroscopy)	
Abscess	
◦ High diffusion signal centrally with relative restricted ADC	
◦ Low diffusion signal in the rim with elevated ADC	
◦ Thin smooth rim of enhancement with thin rim of low T2 signal	
◦ Smooth rim of hypointensity on susceptibility weighted images	
◦ Spectroscopy—amino acids present in the abscess cavity ((valine, leucine and isoleucine; 0.9 ppm), acetate (1.9 ppm), alanine (1.5 ppm), and succinate (2.4 ppm))	
Metastatic disease	
◦ High perfusion in the enhancing mass	
◦ Tend not to occur in the basal ganglia, thalami, or periventricular locations	

### PCNS lymphoma

**Fig. 7:**
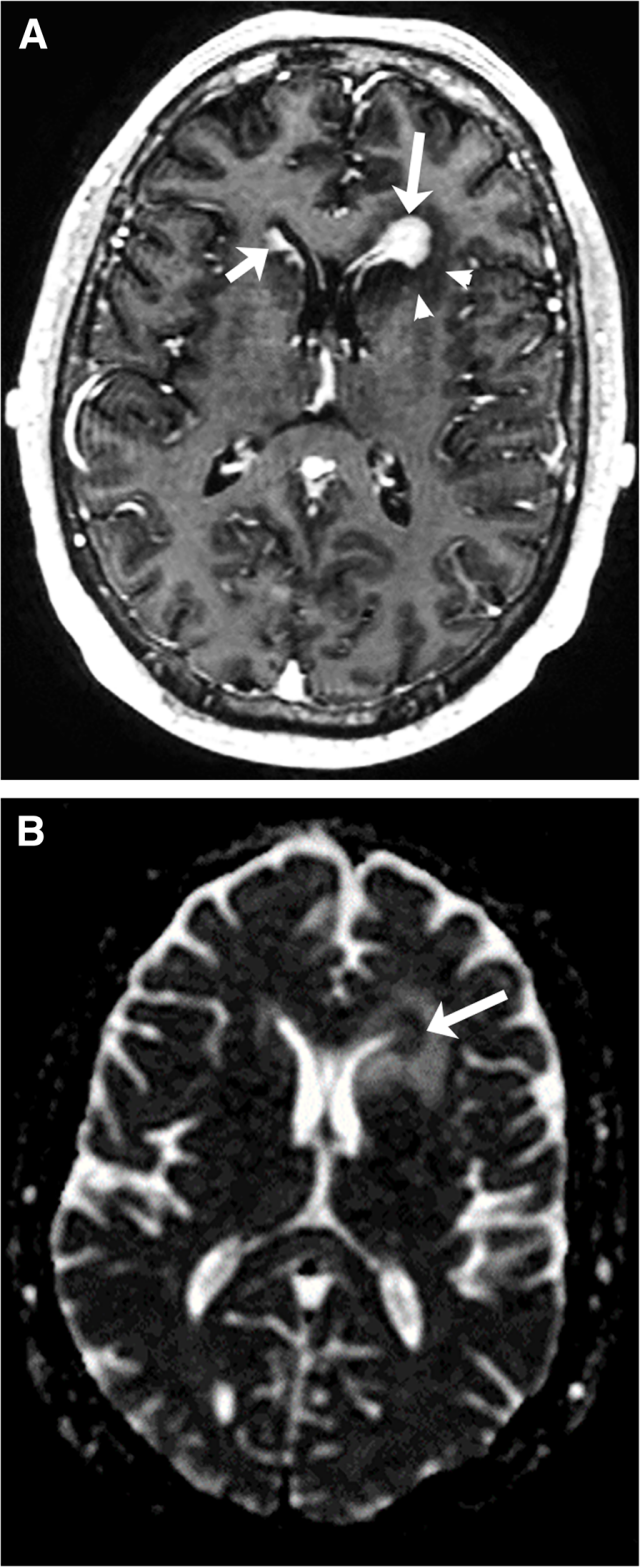
Primary central nervous system lymphoma. T1-weighted image with gadolinium (**a**) shows a diffusely enhanced lesion (long arrow) in the periventricular left frontal horn with mild surrounding vasogenic edema (arrowheads). Also, note the ependymal enhancement along the right frontal horn (short arrow). ADC map (**b**) shows homogeneous diffusion restriction (arrow)

PCNS lymphoma has many similar features to CNS-PTLD but there are a number of differences (Table 1). PCNS lymphoma compared to CNS-PTLD has more diffuse and homogeneous enhancement with well-defined margins of enhancement (Figs. 5 and 7) [14, 25]. Less frequent types of enhancement for PCNS lymphoma are “open-ring-like” enhancement, ring enhancement, and notch enhancement. The lesions tend to involve a CSF or pial surface (80–90%) more commonly than in CNS-PTLD (Figs. 5 and 7). PCNS lymphoma is one of the two main mass lesions that typically involve the corpus callosum, the other lesion being GBM. The lesions in PCNS lymphoma have a predominance of being unifocal but can be multifocal with Sutherland et al. reporting more multifocal cases [25, 26]. The signal intensities on T1 and T2 have been reported as similar to CNS-PTLD [26]. When unifocal, the lesions were significantly larger [25]. The frontal lobes and corpus callosum are commonly involved and the presence of lesions in the basal ganglia and posterior fossa are infrequent (either isolated or associated with supratentorial disease), contrasting with CNS-PTLD. Leptomeningeal disease is rare but possible and edema is a common finding, both similar to CNS-PTLD. The presence of calcification or hemorrhage is uncommon. In fact, the lack of intratumoral susceptibility signals (ITSS) changes helps differentiate lymphoma from CNS-PTLD, GBM, or metastatic disease [14, 27].

A common signature feature of PCNS lymphoma is marked associated restricted diffusion in the mass lesions secondary to hypercellularity. The ADC values in PCNS lymphoma are typically lower and more homogeneous than in CNS-PTLD [14]. The ADC and FA values measured in the solid enhancing components of lymphoma are significantly decreased compared to GBM [28]. The perfusion of PCNS lymphomas are relatively low with reported rCBVs 1.29 ± 0.18 to 2.74 ± 0.87 [29–31]. The variation in the rCBVs measured likely relates to the analysis technique but the rCBV values are consistently found to be significantly lower than in high-grade gliomas. The signal recover of the perfusion curve is also significantly higher (rising above baseline) in PCNS lymphoma than in high-grade gliomas [31–33]. This leakage pattern does not prove PCNS lymphoma but it is an important diagnostic clue [33]. Spectroscopy can show the presence of lipid and/or lactate, increased Cho/Cr, and decreased NAA/Cho [34]. However, this pattern can be seen with GBM and metastatic disease. A massively elevated lipid peak and a markedly elevated Cho/Cr ratio has been found to be more suggestive of PCNS lymphoma than glioma [35]. The spectral pattern might help to differentiate from abscesses which often demonstrate a number of amino acids peaks not seen in PCNS lymphoma.

### Glioblastoma

Glioblastoma (GBM) WHO grade IV is the most common primary intraparenchymal tumor, is typically ring enhancing, and can have a similar appearance to CNS-PTLD. GBM mostly occurs in the cerebral hemispheres and rarely in the posterior fossa or spinal cord. GBMs are a common mass lesion to involve the corpus callosum and this is a less common characteristic of CNS-PTLD. Predominantly, GBMs presents as single masses but multifocal or multicentric disease can occur around 33% of the time. Typically, GBM presents as a large heterogeneous mass with central necrosis and concave shaggy enhancing margin. GBMs occur in all age groups but has a peak incidence in the 7th and 8th decades. GBM is a diagnostic consideration for many ring-enhancing brain lesions with surrounding edema as seen with CNS-PTLD. However, GBM can infrequently present as a solid enhancing mass with necrosis only present at pathological analysis. ITSS signal changes are often found in GBMs and could represent microhemorrhages or calcifications [27]. Massive hemorrhage can occur and this will obscure the underlying tumor.

**Fig. 8:**
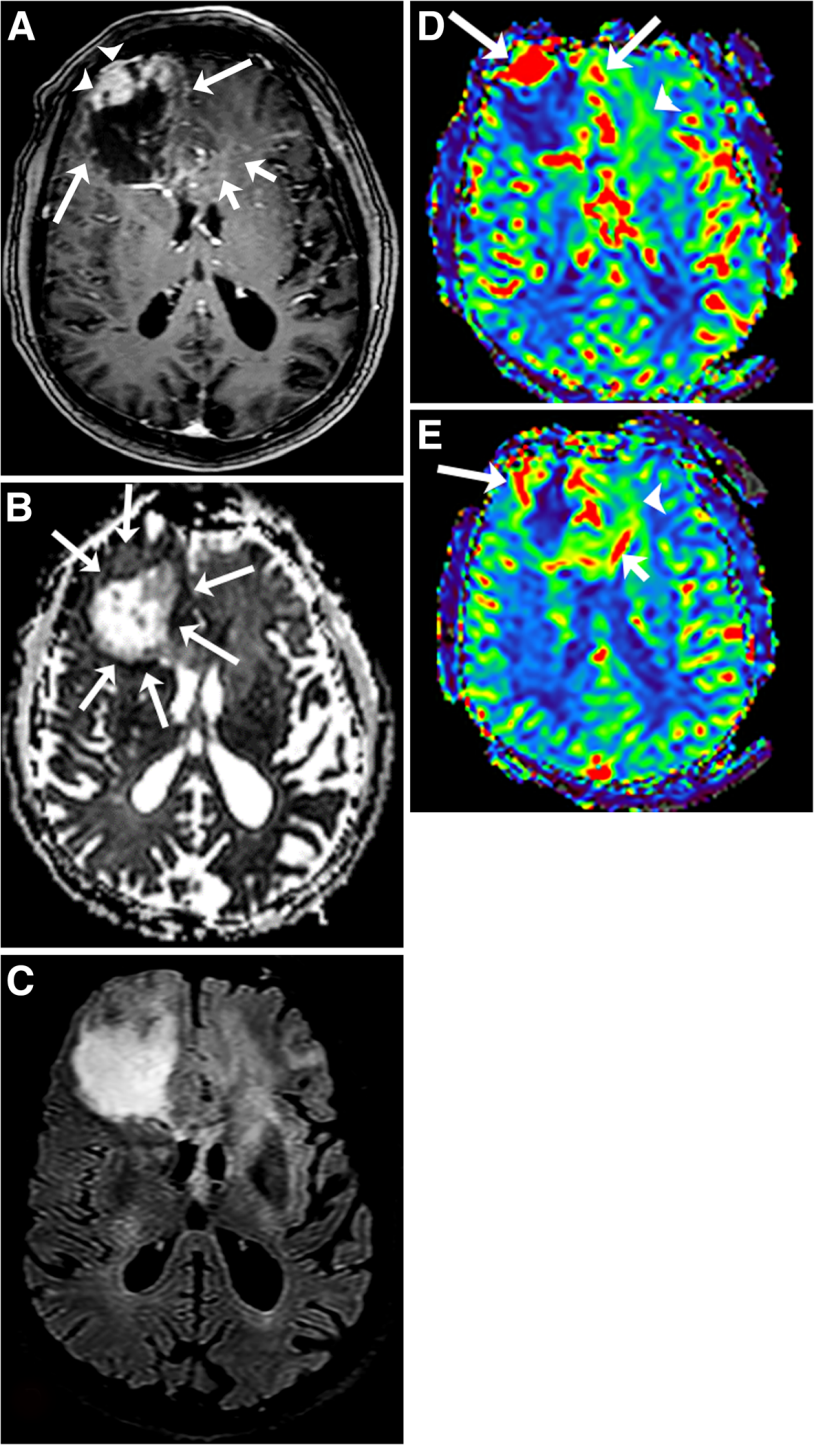
Glioblastoma. T1-weighted image with gadolinium (**a**) shows an irregular rim-enhancing mass with thick (arrowheads) and thin (long arrows) areas of enhancement in the right frontal lobe. The mass extends into the genu of the corpus callosum and left frontal lobe with minimal irregular enhancement (short arrows). ADC map (**b**) demonstrates predominant elevated ADC within the necrotic mass. The rim of the mass has minimal decreased ADC values (arrows). T2 FLAIR (**c**) shows the necrotic right frontal lobe mass, enlargement of the genu of the corpus callosum and diffuse mild T2 high signal abnormality in the left frontal lobe. On CBV map (**d**, **e**), there is associated increased perfusion in the rim of the mass (long arrows) in the right frontal lobe, corpus callosum (short arrow), and to a lesser degree in the left frontal lobe (arrowheads)

The solid component of GBM in general demonstrates elevated rCBV, and elevated ADC values [32, 36]. Where there is hypercellularity, ADC values will be decreased. The FA and ADC values in lymphoma are usually lower than in GBM, likely reflecting greater relative cellularity of PCNS lymphoma [28]. A leakage pattern of the contrast enhancement curve occurs with GBM but not as frequently as in PCNS lymphoma. MR spectroscopy in GBM usually demonstrates elevated lipids and/or lactate, elevated Cho/Cr ratios, and low NAA/Cho ratios. Important imaging findings that favor a diagnosis of GBM are biomarkers that are positive outside of the area of enhancement (the peritumoral area). The ADC might be restricted, the CBV elevated, and the spectroscopy values can indicate tumor in areas outside of enhancing abnormalities in GBM given its infiltrative nature (Fig. 8) [37–40]. The peritumoral area of GBM has been found to have decreased ADC compared to PCNS lymphoma [40]. This relationship would be expected to be present in CNS-PTLD.

### Abscess

**Fig. 9:**
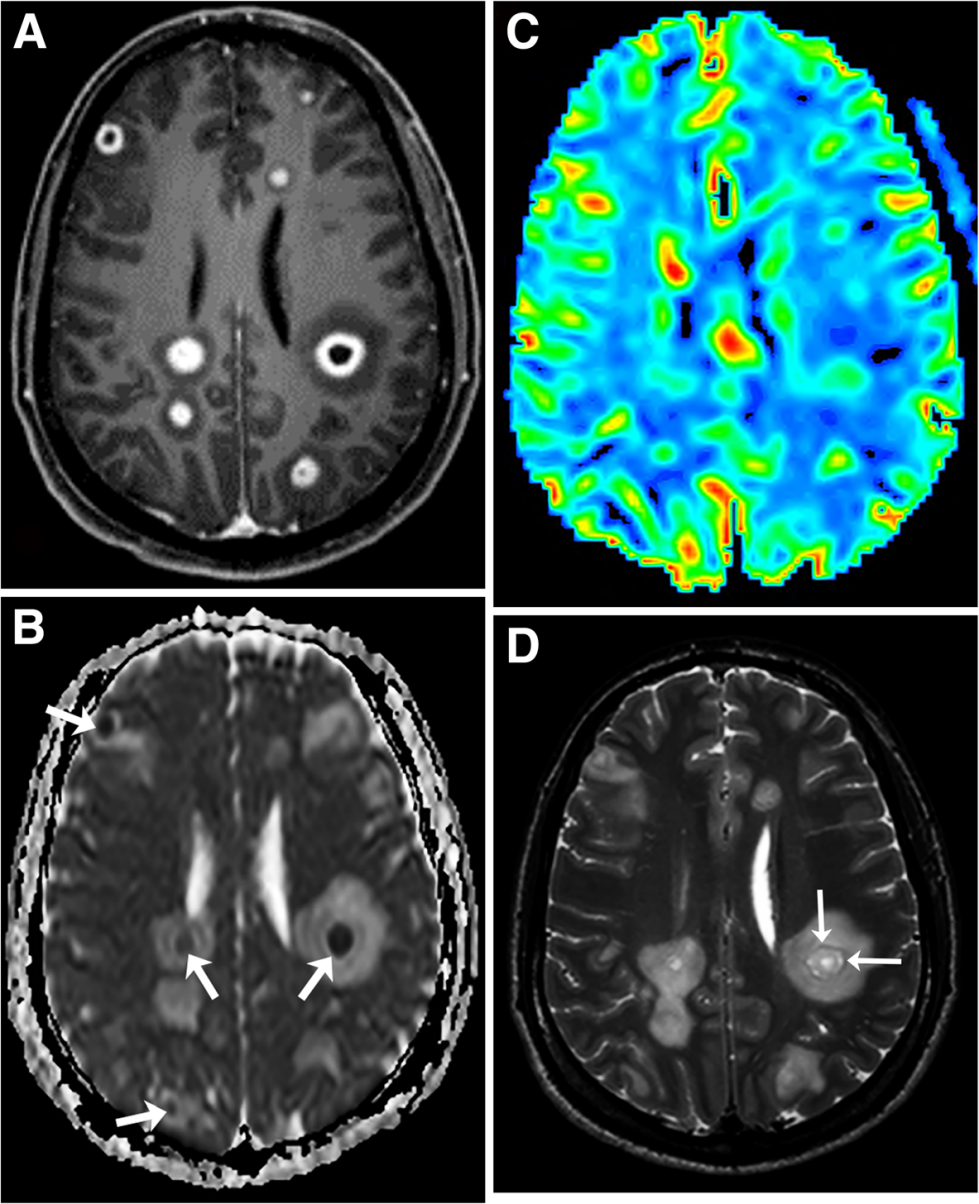
Streptococcus brain abscesses. T1-weighted image with gadolinium (**a**) demonstrates multiple lesions with rim enhancement and surrounding vasogenic edema. The central core of the lesions restrict strongly (arrows) on the ADC map (**b**). The rims are not restricted. CBV map (**c**) shows mild elevated perfusion in the lesions, but no high perfusion in the surrounding area. Note the T2 hypointense rim (arrows) (**d**)

CNS pyogenic abscesses if hematogenously spread occur supratentorially and in subcortical locations [41]. When abscess occurs secondary to direct extension, the abscess is adjacent to where it originated such as the paranasal sinuses or the temporal bone. Abscesses are more commonly single than multiple, which is the opposite of CNS-PTLD lesions. However, abscesses are more likely to be multiple in the immunosuppressed patient. CNS pyogenic abscesses are round or oval with a thin smooth rim of enhancement. The characteristics of the enhancing wall can help differentiate an abscess from CNS-PTLD in which the walls are thicker and more irregular. The abscess capsule may be seen as a thin band of T2 hypointensity that is relatively specific for abscess (Fig. 9) [41]. Susceptibility-weighted imaging in a pyogenic abscess can demonstrate a thin rim of hypointensity or dual-rim sign but in fungal abscesses, one finds a prominent peripheral rim or central susceptibility effects [42, 43]. These appearances are different than for CNS-PTLD which demonstrates a peripheral pattern of punctate susceptibility-weighted hypointensities.

DWI and ADC analysis of the abscess cavity are also very useful [44–47]. Abscesses tend to have cavities of relative restricted ADC and high diffusion signal, whereas the necrotic cavities of tumors have higher ADC values [47]. The opposite is true for the rims of these lesions with higher ADC values in the rims of abscesses and lower ADC values in the rims of tumors. The diffusion characteristics of the rims of abscesses and tumors offer the better diagnostic differentiation between these entities. The reason is even though the central cavities of tumors usually have lower signal on DWI and elevated ADC values, the presence of hemorrhage and the debris within necrotic components of tumors can result in elevated DWI signal and lower ADC values that mimic the appearance of an abscess.

On PWI, abscesses typically have mildly elevated CBV [44, 46, 48, 49]. This makes PWI less useful for differentiating an abscess from lymphoma or likely CNS-PTLD. The abscess MR spectrum does not have elevated choline but has elevated amino acids from 0.9–2.4 ppm ((valine, leucine and isoleucine; 0.9 ppm), acetate (1.9 ppm), alanine (1.5 ppm), and succinate (2.4 ppm)) [46]. This spectrum when present helps identify an abscess cavity.

**Fig. 10:**
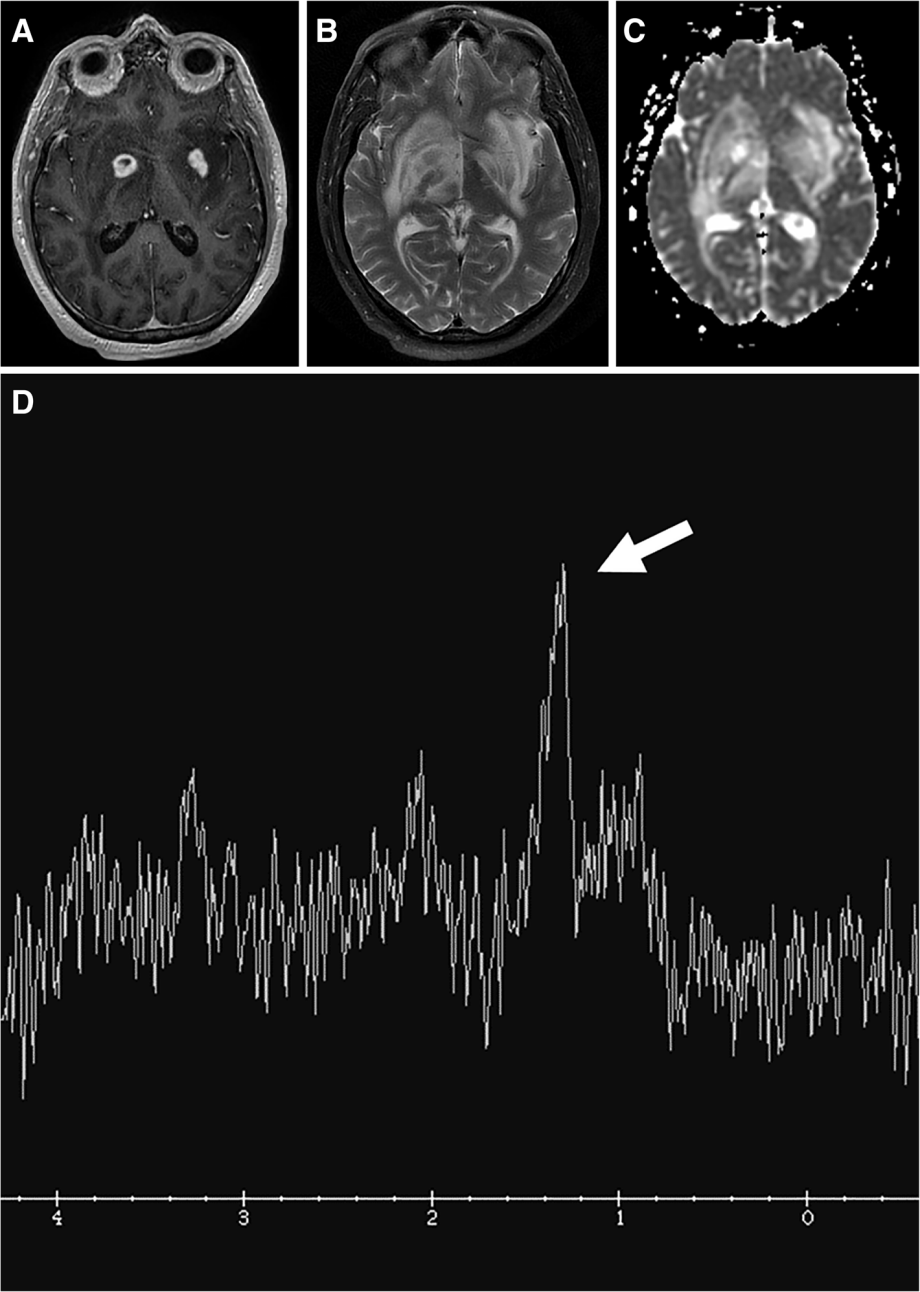
Cerebral toxoplasmosis. T1-weighted image with gadolinium (**a**) shows multiple ring and nodular-enhancing masses in the basal ganglia. T2-weighted image (**b**) shows extensive edema. No obvious restricted diffusion in the lesions is present on the ADC map (**c**). Single voxel spectroscopy (TE = 35) (**d**) demonstrates elevated lipid/lactate peak (arrow) and all other peaks are decreased

Neurotoxoplasmosis, an opportunistic infection, is the most common cause of cerebral abscess in immunocompromised patients. Like CNS-PTLD, cerebral toxoplasmosis tends to present as multiple lesions in the basal ganglia and at the corticomedullary junctions. These lesions are characterized by a ring or nodular enhancement, so it must be considered in a differential diagnosis of CNS-PTLD. Toxoplasmosis on MR spectroscopy often shows increased lactate and lipids and reduced Cho, Cr, and NAA (Fig. 10) [50].

### Metastatic disease

The imaging characteristics of metastatic disease overlap significantly with CNS-PTLD. Many cases of metastatic disease in the brain are ring enhancing with multiple lobar lesions but single metastatic brain lesions are not uncommon. These features are similar to CNS-PTLD. However, metastatic disease is not as commonly present in the basal ganglia, thalami, and periventricular locations as CNS-PTLD. CNS-PTLD and metastatic disease both often have ring enhancement that is heterogeneous. PCNS lymphoma is a usually a more solid homogeneously enhancing disease process.

**Fig. 11:**
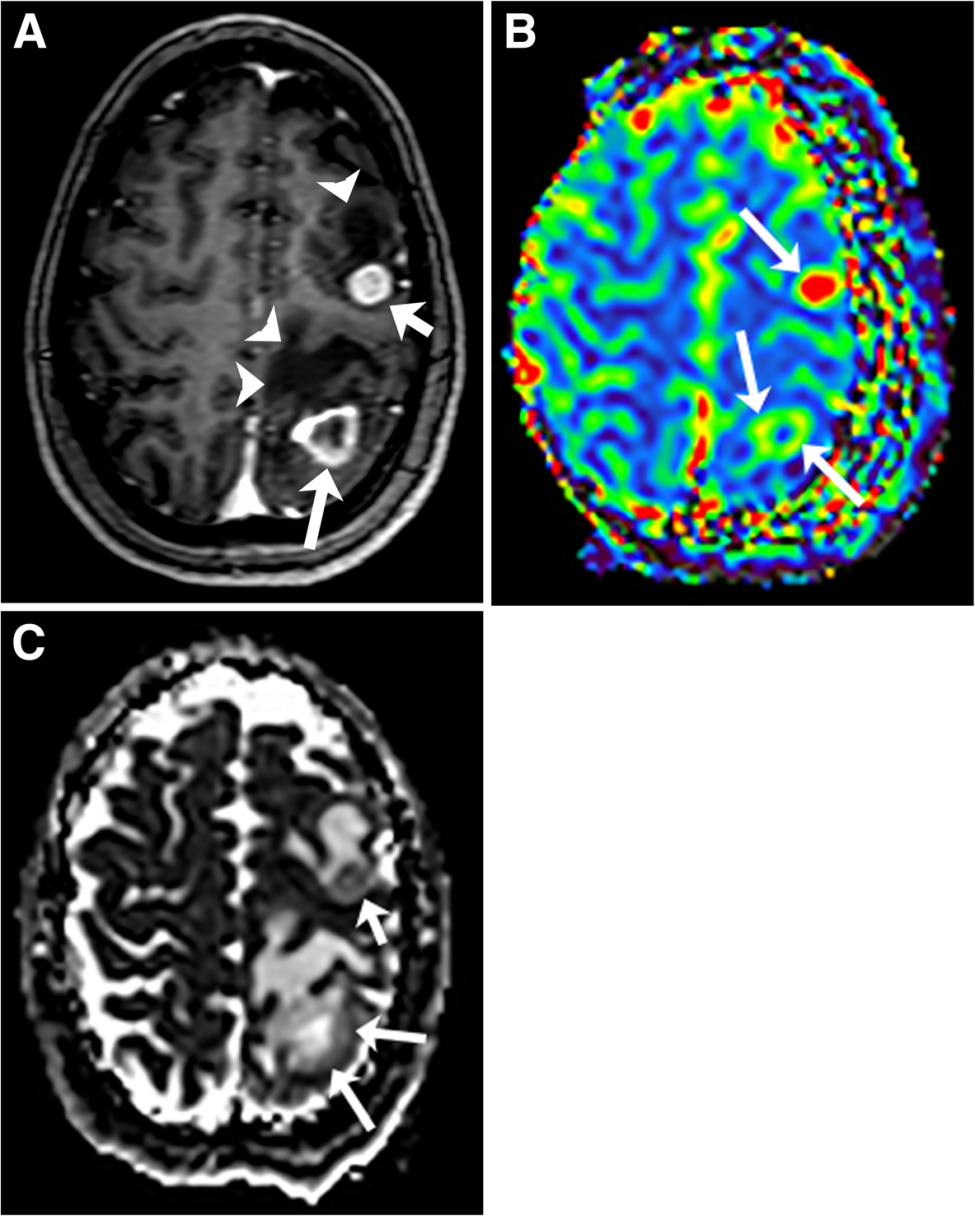
Metastasis in a patient with lung adenocarcinoma. T1-weighted image with gadolinium (**a**) demonstrates a ring-enhancing left parietal lobe mass (long arrow) and a solidly enhancing left frontal lobe mass (short arrow). Extensive vasogenic edema is around each of these lesions (arrowheads). On CBV map (**b**), the CBV is elevated in the enhancing components (arrows). No increased peri-tumoral enhancement or elevated perfusion is present. ADC map (**c**) shows minimally decreased ADC in the parietal mass (long arrows) and mildly decreased ADC in the frontal mass (short arrow)

The DWI and spectral appearance of metastatic disease and CNS-PTLD are expected to be similar with no comparisons having been made directly between these disease processes. Metastatic disease has tumor spectroscopy findings of decreased NAA/Cho, elevated Cho/Cr, and presence of lipid and lactate [39]. Lymphomas do tend to have more restricted diffusion than metastatic disease. However, a hypercellular metastatic lesion would likely have lower ADC values that could be similar to lymphoma. PWI should likely help to differentiate metastatic disease since metastatic lesions usually have high perfusion and lymphomas do not. This is expected to hold true for CNS-PTLD but perfusion data on CNS-PTLD is very limited (Fig. 3) [22]. Perfusion, DWI/ADC, and spectroscopy analysis can be confounded by the presence of extensive necrosis or hemorrhage obscuring the metastatic disease. Both CNS metastatic disease and CNS-PTLD should not have peritumural changes that have characteristics of tumor such as seen with GBM (Fig. 11) [37–40].

## Conclusion

CNS-PTLD is a complicated disease process that affects immunosuppressed post-transplant individuals. Being able to accurately help differentiate this diagnosis through imaging is vital and helps optimize making an early diagnosis (Tables 1 and 2). Given the rarity of the diagnosis, the consideration of CNS-PTLD is often lacking on initial imaging interpretations. Advanced imaging characteristics likely will help to diagnosis CNS-PTLD but our review of the literature demonstrates a paucity of information pertaining to these techniques. CNS-PTLD shares imaging characteristics with multiple other disease processes including PCNS lymphoma, GBM, metastatic disease, abscess, and other infections. Even though definitive diagnosis will often come down to biopsy, imaging characteristics will help distinguish the disease processes.
